# Triple Viral Respiratory Co-Infection With Respiratory Syncytial Virus (RSV), Human Metapneumovirus (HMPV), and Influenza A (H1N1) Leading to Acute Respiratory Distress Syndrome (ARDS) in an Infant: A Case Report

**DOI:** 10.7759/cureus.108099

**Published:** 2026-05-01

**Authors:** Mohammed Hatimi, Nora Touyar, Ghizlane El Amin, Amal Zouaki, Soukayna Jebbar, Saad Elharrak, Aziza Bentalha, Selma Ech-Cherif El Kettani, Hakima Kabbaj

**Affiliations:** 1 Central Virology Laboratory, Specialty Hospital - Ibn Sina University Hospital, Rabat, MAR; 2 Faculty of Medicine and Pharmacy, University of Mohammed V, Rabat, MAR; 3 Pediatric Intensive Care Unit, Children's Hospital - Ibn Sina University Hospital, Rabat, MAR

**Keywords:** cycle threshold, human metapneumovirus (hmpv), influenza a(h1n1) pdm09, multiplex pcr, respiratory syncytial virus (rsv), viral co-infection

## Abstract

Viral respiratory infections are a major cause of morbidity in pediatrics. We report a rare case of triple infection with respiratory syncytial virus (RSV), human metapneumovirus (HMPV), and influenza A (H1N1) in a previously healthy nine-month-old infant, complicated by acute respiratory distress syndrome (ARDS). The patient presented with severe respiratory distress, elevated inflammatory markers, and bilateral alveolar infiltrates, requiring non-invasive ventilation. A point-of-care multiplex PCR using the BioFire® Respiratory Panel 2.1 plus (RP2.1plus; bioMérieux, Marcy-l'Étoile, France) detected all three viruses. Confirmatory testing with the Xpert® Xpress Flu/RSV quadriplex PCR (Cepheid, Sunnyvale, CA) showed low cycle threshold (Ct) values for RSV and H1N1. Despite antiviral treatment, the patient’s condition worsened, requiring invasive mechanical ventilation for 60 days. This case highlights the rarity and diagnostic complexity of triple respiratory viral co-infection and underscores the role of multiplex PCR in rapid pathogen detection. The interpretation of Ct values may provide additional diagnostic information but should be considered within the clinical context.

## Introduction

Viral respiratory infections represent a major public health concern in the pediatric population due to their high transmissibility and associated morbidity [1-3]. Respiratory syncytial virus (RSV) and human metapneumovirus (HMPV), both members of the *Pneumoviridae *family, are leading causes of bronchiolitis and lower respiratory tract infections in infants [2,4]. Influenza A (H1N1) virus, a member of the *Orthomyxoviridae *family, causes seasonal epidemics and may also lead to severe respiratory complications in young children [1,5]. Viral co-infections are increasingly recognized in pediatric respiratory infections; however, their clinical impact remains incompletely understood. Reports of triple viral co-infection involving these pathogens remain extremely limited. We report a rare case of triple infection with RSV, HMPV, and H1N1 in an infant, complicated by acute respiratory distress syndrome (ARDS), highlighting the diagnostic and clinical challenges associated with such co-infections [6,7].

## Case presentation

**Figure 1:**
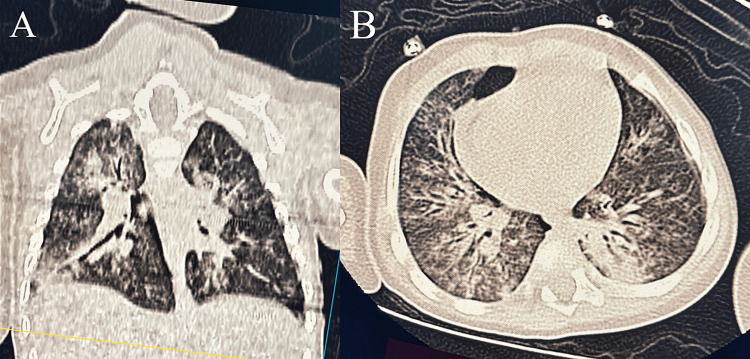
Coronal and axial views of the C+ chest CT. (A) Coronal CT image showing bilateral diffuse pulmonary infiltrates, consolidation, and peribronchovascular thickening. (B) Axial CT image showing bilateral diffuse confluent ground-glass nodules.

A nine-month-old male infant, with no prior medical history and up-to-date vaccinations according to the Moroccan national immunization schedule, presented with progressive respiratory distress associated with rhinorrhea, dry cough, fever, and feeding difficulties evolving over several days. On admission, he was tachypneic and tachycardic, with signs of increased work of breathing and bilateral wheezing on auscultation. Chest X-ray demonstrated bilateral bronchial wall thickening consistent with bronchiolitis. He was initially admitted with a presumed diagnosis of superinfected viral bronchiolitis and started on amoxicillin-clavulanic acid. After three days without clinical improvement and progressive respiratory deterioration, he was transferred to the pediatric intensive care unit (PICU). At that time, he presented with severe respiratory distress (Silverman-Andersen score: 8/10) [8], requiring high-flow oxygen therapy (10 L/min), continuous nebulized bronchodilators, and intermittent non-invasive ventilation. A contrast-enhanced chest CT scan, performed due to clinical-radiological discordance, revealed bilateral consolidations predominantly involving the basal regions and the right upper lobe, diffuse ground-glass opacities, and peribronchovascular thickening, consistent with bilateral infectious pneumonia with features suggestive of early alveolar edema (Figure 1).

Initial laboratory workup showed severe inflammatory syndrome with neutrophil-predominant hyperleukocytosis (22,000 cells/mm³), elevated C-reactive protein (210 mg/L), and procalcitonin (4.7 µg/L). Arterial blood gas analysis under oxygen therapy (FiO₂ 0.57) showed a PaO₂ of 84 mmHg, a PaCO₂ of 46 mmHg, and a PaO₂/FiO₂ ratio of 147 (Table 1).

**Table 1:** Initial laboratory findings

Parameter	Patient Value	Reference Range
White blood cell count	22,000 cells/mm³	5,000–15,000 cells/mm³ (infant)
Neutrophils	Predominant	20%–45%
C-reactive protein (CRP)	210 mg/L	<5 mg/L
Procalcitonin	4.7 µg/L	<0.5 µg/L
PaO₂	84 mmHg (FiO_2_ 0.57)	80–100 mmHg
PaCO₂	46 mmHg	35–45 mmHg
PaO₂/FiO₂ ratio	147	>300

The etiological workup was completed using a BioFire® FilmArray multiplex respiratory panel 2.1 plus (RP2.1plus; bioMérieux, Marcy-l'Étoile, France) performed on a nasopharyngeal swab, which detected RSV, HMPV, and H1N1 (pdm2009 strain). A complementary Xpert® Xpress CoV-2/Flu/RSV quadruplex PCR (Cepheid, Sunnyvale, CA) on the same sample was also positive, with cycle threshold (Ct) values of 20.5 for RSV and 21.3 for H1N1. The patient was subsequently started on oseltamivir (30 mg every 12 hours) for a total of five days without improvement. The clinical course progressed to ARDS, defined according to the Berlin criteria by an acute onset of respiratory failure within one week, a PaO₂/FiO₂ ratio deteriorating below 100 mmHg under positive end-expiratory pressure (PEEP) of 8 cm H₂O, bilateral pulmonary opacities on imaging, and a non-cardiogenic origin. The patient required invasive mechanical ventilation. Repeated extubation attempts failed due to severe desaturation, necessitating tracheostomy. Gradual respiratory improvement allowed decannulation after 60 days of ventilation, and the patient was eventually discharged after a three-month PICU stay.

## Discussion

Viral respiratory infections account for 30%-50% of pediatric consultations and 20%-40% of hospitalizations [1]. RSV, HMPV, and H1N1 are common etiologies of respiratory infections in children, with RSV being the leading cause of viral bronchiolitis in infants [5]. Viral respiratory co-infections have been increasingly recognized; however, reports of triple viral co-infection involving these pathogens remain extremely limited. In particular, the coexistence of RSV, HMPV, and H1N1 is rarely reported in the literature. Available data on the incidence of such co-infections remain limited and heterogeneous [1,2,5]. In our setting, only one case was identified among 2,500 positive multiplex PCR respiratory samples processed at the Central Virology Laboratory (Ibn Sina University Hospital) since the implementation of the FilmArray® system in 2021. The clinical significance of viral co-infections remains controversial. While some studies suggest a potential synergistic pathogenicity leading to higher rates of ICU admission and mechanical ventilation, particularly in RSV-associated co-infections, the evidence remains inconsistent and is largely derived from observational studies with heterogeneous populations [2,3]. In this context, one study reported a higher risk of ICU admission in bronchiolitis cases involving RSV-HMPV co-infection compared to monoviral infections [4], although these findings are based on limited non-randomized data.

In our case, the detection of the three viruses via qualitative molecular techniques such as the FilmArray multiplex respiratory PCR does not necessarily prove their causal contribution to the disease process. Indeed, a positive PCR result does not equate to viral replication or infectivity [6]. This highlights the importance of cautious interpretation of multiplex panels in clinical practice. In this context, the complementary Xpert® Xpress CoV-2/Flu/RSV quadruplex assay confirmed the presence of the viruses and provided an indirect estimation of viral burden through Ct values. However, Ct values are semiquantitative and not standardized across platforms, and therefore remain an imperfect surrogate for infectivity. Notably, human metapneumovirus (HMPV) cycle threshold values were not available, as HMPV was not included in the quadruplex assay and no separate monoplex PCR was performed in our laboratory.

The patient developed ARDS, defined by the Berlin criteria as acute-onset hypoxemia requiring PEEP ≥ 5 cm H₂O, bilateral pulmonary infiltrates, and non-cardiogenic pulmonary edema [9]. ARDS in viral bronchiolitis is multifactorial and involves inflammatory injury, surfactant dysfunction, and increased alveolor-capillary permeability [6]. In our case, no host-related risk factors for severe disease, such as prematurity, chronic cardiopulmonary disease, neuromuscular disorders, or immunodeficiency, were identified [6,7]. This suggests that pathogen-related factors may have contributed to ARDS, including viral co-infection, high viral load, secondary bacterial superinfection, and the absence of specific antiviral treatment for viruses such as RSV and HMPV [7]. In addition, respiratory infections caused by RSV and H1N1 have been associated with severe disease progression, including ARDS [7], which is consistent with the clinical course observed in our case. Although inhaled ribavirin has been used in selected severe RSV infections, its use in our case could be considered; however, its clinical benefit in the context of ARDS remains unestablished [5,7]. Overall, this case highlights the diagnostic complexity of viral co-infections and the challenges in attributing causality when using multiplex molecular testing, particularly in severe pediatric respiratory disease.

## Conclusions

This case describes a rare triple respiratory viral infection, highlighting its infrequent reporting in the literature and the diagnostic complexity of multiple viral detections in a single clinical context. Point-of-care syndromic PCR allowed for rapid detection of the suspected viruses, while quantitative or semiquantitative PCR provided additional diagnostic information through Ct values, which may offer indirect insights into viral burden. This case underscores the utility of multiplex molecular assays in the early identification of respiratory viral pathogens in critically ill pediatric patients. Although molecular detection does not establish causality, Ct-based parameters remain imperfect surrogates that must be interpreted within the clinical context. This report emphasizes the importance of integrating molecular findings with clinical assessment and the need for further studies to clarify the clinical significance of multiple respiratory viral co-infections.
